# Prediction of Protein-protein Interactions on the Basis of Evolutionary Conservation of Protein Functions

**Published:** 2007-08-08

**Authors:** Ekaterina Kotelnikova, Andrey Kalinin, Anton Yuryev, Sergei Maslov

**Affiliations:** 1 Ariadne Genomics Inc. 9430 Key West Ave., Suite 113, Rockville, MD 20850, U.S.A.; 2 Department of Physics, Brookhaven National Laboratory, Upton, New York 11973, U.S.A.

**Keywords:** Protein interactions, prediction, functional evolution, sequence similarity

## Abstract

**Motivation:**

Although a great deal of progress is being made in the development of fast and reliable experimental techniques to extract genome-wide networks of protein-protein and protein-DNA interactions, the sequencing of new genomes proceeds at an even faster rate. That is why there is a considerable need for reliable methods of *in-silico* prediction of protein interaction based solely on sequence similarity information and known interactions from well-studied organisms. This problem can be solved if a dependency exists between sequence similarity and the conservation of the proteins’ functions.

**Results:**

In this paper, we introduce a novel probabilistic method for prediction of protein-protein interactions using a new empirical probabilistic formula describing the loss of interactions between homologous proteins during the course of evolution. This formula describes an evolutional process quite similar to the process of the Earth’s population growth. In addition, our method favors predictions confirmed by several interacting pairs over predictions coming from a single interacting pair. Our approach is useful in working with “noisy” data such as those coming from high-throughput experiments. We have generated predictions for five “model” organisms: *H. sapiens, D. melanogaster, C. elegans, A. thaliana, and S. cerevisiae* and evaluated the quality of these predictions.

## Introduction

*In-silico* methods are widely used to transfer knowledge about protein-protein interaction networks within the organism or between different model organisms, and several general approaches to this task exist. For well-studied model organisms, it is common to use different sources of information which, in addition to sequence homology, include Gene Ontology annotations, localization databases, and other sources to predict and validate protein-protein interactions (Tan et al. 2004; Huang et al. 2004; Ben-Hur and Nobel, 2005; Kemmer et al. 2005; von Mering C et al. 2005). However, not all these data are reliable or easily available for some genomes. That is why it is important to optimize methods of interaction prediction based solely on the most common and reliable information - protein sequence similarity.

A number of existing methods already allow the prediction of protein-protein interactions using sequence similarity. They include domain-based methods (Sprinzak and Margalit, 2001), Bayesian network models (Deng et al. 2002), pairwise sequence kernels (Ben-Hur and Noble, 2005), and co-evolution of interacting proteins (Ramani and Marcotte, 2003). In general, each pair of proteins A′ and B′ that are predicted to interact with each other is given a score S(A′, B′), which needs to be optimized for maximal reliability. This optimization could be subdivided into three separate problems:

1. The first problem is finding an optimal mathematical expression for individual prediction scores s_i_(A_i_, B_i_ – >A′, B′), which is the probabilistic measure of predicted interaction A’ – B’ to be true, based on the knowledge that a known interacting protein pair A_i_ – B_i_ exists in the training set, where A’ is homologous to A_i_ and B’ is homologous to B_i_.
2. The second problem is the definition of the combined score S(A′, B′), which is a rule for construction of a final score based on several individual scores s_i_(A_i_, B_i_ – >A′, B′). Until now, most of the considered prediction scores defined the combined score as equal to the *maximum* of individual scores (e.g. Yu et al. 2004).
3. The third problem is the definition of “characteristic protein family size,” which defines a number of “individual scores” that should be taken into account when one calculates the final score for the predicted A’ – B’ pair. This problem exists in cases of certain combined scores where it is necessary to avoid good final scores that have resulted from many bad individual predictions.

Our method is based on novel approaches to all of the problems previously mentioned: 1) our individual score s_i_(A_i_, B_i_ – >A′, B′) is based on an empirically derived probabilistic formula instead of ad hoc assumptions, and 2) the combined score is taken as the combination of individual scores. Therefore, in general, we score the predictions confirmed independently by several known interactions higher than the predictions based on a single known interaction. The cumulative effect for predicting protein-protein interactions was independently used in Jonsson et al. 2006. Finally, in 3) we carefully determine the “characteristic protein family size” in order not to miss some important prediction events. We show that these approaches provide significant improvement for sequence-based prediction methods, especially for noisy interaction datasets, such as those coming from high-throughput experiments.

## Materials and Methods

### Main terms

- *Interparalogs* or *generalized interparalogs.* If interacting proteins A and B have paralogs A′ and B′ which also interact, we will call the pairs A – B and A′ – B′ interparalogs. There could be several interparalog pairs within the organism, which can be called generalized interparalogs.
- *Interologs*. If two interacting proteins A and B have orthologs A′ and B′ that also interact, according to Walhout et al. 2000, we call the pairs A – B and A′ – B′ interologs.
- *Generalized interologs*. Using the definition from Yu et al. (2004), if A – B and A′ – B′ are interologs, we consider generalized interologs to be all possible interactions between protein families {A′} and {B′}, i.e. interactions between all A and B homologs in the target organism.

### Main assumptions

To predict interactions on the basis of protein sequence similarities, the following assumptions have been made:

- We use protein sequence similarity as the measure of homology between two proteins. The similarity between two protein sequences is taken from the similarity score calculated by the BLAST (Altschul et al. 1990) program using the standard BLOSUM62 substitution matrix in the manner as described in (Ispolatov et al. 2005).
- We use only the pairs of orthologous proteins consisting of reciprocal best matching homologs in both organisms, i.e. not more than one ortholog of a specific protein per each organism.

### Training sets

The protein interaction data for all species were obtained from the ResNet databases of Biological Association Network available from Ariadne Genomics (http://www.ariadnegenomics.com/). The databases for *Homo sapiens*, baker’s yeast *Saccharomyces cerevisiae*, the nematode worm *Caenorhabditis elegans*, the plant *Arabidopsis thaliana*, and the fruit fly *Drosophila melanogaster* were constructed by combining the data from published high-throughput experiments, publicly available interaction databases such as BIND and EntrezGene with the literature data extracted using MedScan technology from organism-specific PubMed abstracts and full-text articles. For more details about the construction of these databases, please refer to the PathwayStudio manual: http://www.ariadnegenomics.com/products/pathway.html

For evaluation of prediction quality, the following “golden sets” were used:

#### Saccharomyces cerevisiae:

- Golden positives—MIPS data (Mewes et al. 2000), taken from http://interolog.gersteinlab.org (Yu et al. 2004) for comparison purposes.
- Golden negatives were taken from the same source, http://interolog.gersteinlab.org (Yu et al. 2004).

#### Homo sapiens:

- Golden positives for EntrezGene interactions from http://www.ncbi.nlm.nih.gov/entrez/query.fcgi?db=gene.
- As a golden negative set, the random combinations of non-interacting proteins were used. We set the size of the negative set to be the same as the size of the golden positive set.

#### Arabidopsis thaliana, Caenorhabditis elegans, Drosophila melanogaster:

- Golden positives from ResNet data. As long as a self-prediction of the interactions is not allowed, the training dataset could be the same as evaluation set.
- Golden negatives were not used due to the lack of reliable information about full interaction networks.

## Evolution and Score Definition

### Evolution of protein functions and definition of the individual score

The problem of the individual score definition for predicted interactions can be formulated as follows: given the measure of sequence similarity between knowing interacting proteins and their homologs, it is necessary to have a measure of similarity between the original and the target pairs of proteins in such a way that this measure indicates how likely the interaction is for a target pair of proteins.

The sequence-based score definitions have been discussed elsewhere (Ben-Hur and Nobel, 2005). They include: the geometric mean of individual similarities between homologous proteins or minimum of these similarities (Yu et al. 2004), use of various pairwise sequence kernels (Ben-Hur and Nobel, 2005; Martin et al. 2005), or Bayesian networks. All of these methods do not use any empirical data in order to construct the model. In this case, we attempted to incorporate knowledge about the evolution of the protein-protein interaction network to construct the individual prediction score. We used the modified procedure described in (Maslov et al. 2004) to plot the percentage of physical interaction partners shared by a pair of paralogous proteins as a function of their amino acid sequence similarity.

For a pair of paralogs A and A′, the overlap is defined as the number of their common binding partners in the network. This value is normalized by the total number of binding partners for two paralogs, resulting in the value *p*_AA′_, which can be treated as a probability for protein A′ to keep the same interaction partner as protein A. We refer to the value 1– *p*_AA′_ as “functional distance” between two homologous proteins. This is a probability to not observe an interaction between proteins A′ and B, given that the protein B interacts with protein A averaged over different B’s.

**Figure 1.**
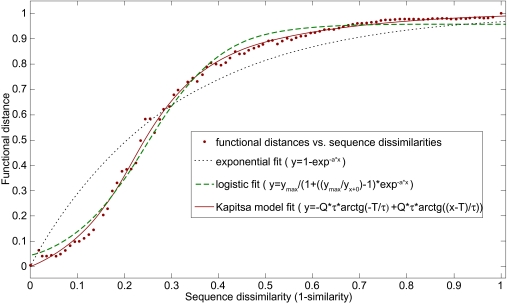
The empirically measured average probability to not observe an interaction between proteins A′ and B (functional distance) in *Homo sapiens* given that protein B interacts with protein A paralogous to protein A′ plotted as a function of sequence dissimilarity, calculated as 1- sim(A,A′), where sim(A,A′) is amino acid sequence similarity between A and A’ paralogs. Different lines correspond to different fitting models: exponential fit (dotted line), logistic fit (dashed line), and Kapitza fit (solid line).

The dependency between the average “functional distance” *F* _AA′_ and sequence similarity between paralogous proteins for *Homo sapiens* network is shown in Figure 1. To approximate this dependency, several different formulas could be applied. In the original paper (Maslov et al. 2004) the exponential fit

(1) $$F_{\text{A}{\text{A}}^{\prime }}\;=\;1\;-\;p_{\text{A}{\text{A}}^{\prime }}\;\approx \;1\;-\;e^{-\text{a}*\text{x}(\text{A},\;{\text{A}}^{\prime })}$$

was used, which does not take into account the s-shape of the curve. The alternative approximation is a logistic s-shaped formula:

(2) $$\begin{array}{ll} F_{\text{A}{\text{A}}^{\prime }}\;= & 1\;-\;p_{\text{A}{\text{A}}^{\prime }}\;\approx \;F_{\text{max}}/\\ & (1\;+\;(F_{\text{max}}/F_{0}\;-\;1)*e^{-a*\text{x}(\text{A},\;{\text{A}}^{\prime })} \end{array}$$

However, we found that the best approximation is achieved with the function:

(3) $$\begin{array}{ll} F_{\text{A}{\text{A}}^{\prime }}\;= & 1\;-\;p_{\text{A}{\text{A}}^{\prime }}\;\approx \;,\;\approx \;-\;\text{Q}*\;\tau *\;arctg\;(-\text{T}/\tau )\\ & +\;\text{Q}*\;\tau *\;arctg((x(\text{A},\;{\text{A}}^{\prime })\;-\;\text{T})/\tau ) \end{array}$$

This formula had been introduced by S.P. Kapitza in the context of population studies (S.P. Kapitza 1996). Here, Q = C* τ *^2^* is the maximum growth rate, τ is the small parameter and T is the time (or time analog) when the system changes the rate of growth.

**Figure 2.**
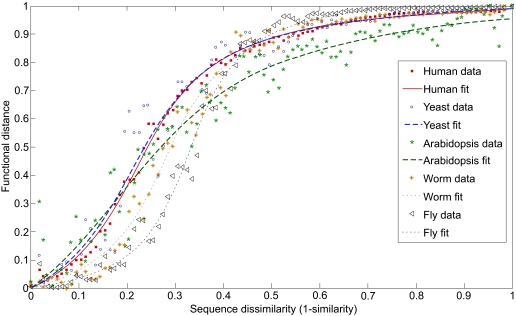
The empirically measured probability to not observe an interaction between proteins A′ and B, given that protein B interacts with protein A paralogous to the protein A′ plotted as a function of sequence dissimilarity, calculated as 1– sim(A,A′), where sim(A,A′) is amino acid sequence similarity between A and A’ paralogs. Different symbols correspond to different model organisms: yeast (o), worm (*), fly (triangle), arabidopsis (star), and human (square). Different lines are fits corresponding to Kapitza models.

Similar data for all five studied organisms are shown in Figure 2. While the approximation curves vary slightly for different species, the coefficients found by the least square method (with 95% confidence bounds) lies within the following intervals:

(4) $$\begin{array}{l} \text{Q}\;=\;2.1\;÷\;4.5\\ \text{T}\;=\;0.19\;÷\;0.33\\ \tau \;=\;-0.12\;÷\;0.22 \end{array}$$

The main parameter here is T, which corresponds to the characteristic sequence similarity (1–T). This parameter could be used as a threshold value for the functional conservation of the proteins during the evolution. For the proteins within one organism, this parameter leads to a similarity threshold value of 0.7–0.8 (70–80%), which aligns with a common sense approach.

**Figure 3.**
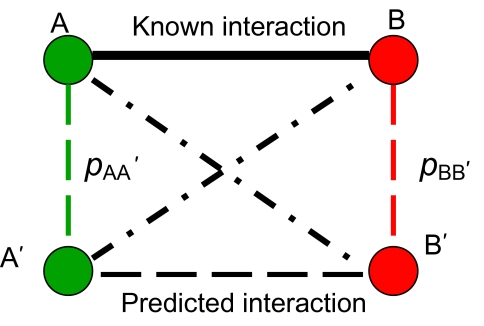
The illustration of the logic behind our expression for the individual similarity score: *pi*(*A,B –>A′,B′*)= *p*AA′*pBB′. Here, *p*AA′ is the probability of detecting interaction A’—B, given the fact that A interacts with B. *p*BB′ is the probability of detecting interaction A’—B’, given the fact that A’ interacts with B. Thus, the probability of detecting A’—B’, given that A—B is *p*AA′ *p*BB′.

If there is an interaction between proteins A_i_ and B_i_, one can define the probability *p*_i_ (individual score) of the interaction between their respective paralogs A′ and B′ as a product of the probabilities *p*_A_i_A′_ and *p*_BiB′_. (See the diagram in Figure 3).

(5) $$p_{i}(A^{\prime },\;B^{\prime })\;=\;p_{A_{i}A^{\prime }}*\;p_{B_{i}B^{\prime }}\;=\;(1\;-\;F_{A_{i}A^{\prime }})\;(1\;-\;F_{B_{i}B^{\prime }})$$

This value should be incorporated into the final prediction score.

**Figure 4.**
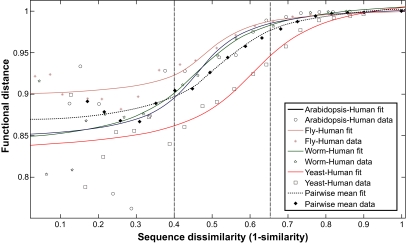
The functional distance between Ax protein from organism X and Ay’ protein from organism Y was calculated as the average empirically measured probability to not observe an interaction between proteins Ay′ and By in organism Y, given that protein Bx (which is a the only true ortholog of By from organism X) interacts with protein Ax′ paralogous to the protein Ax (which is the only true ortholog of Ay). The plot shows how the functional distance depends on the sequence dissimilarity calculated as 1– sim(Ax,Ay′), where sim(Ax,Ay′) is the amino acid sequence similarity between Ax and Ay’ proteins. Different symbols correspond to different pairs of model organisms: arabidopsis -human (circle), fly-human (*), worm-human (five-sided star), yeast-human (square), averaged data over all pairs (diamond). Other pairwise combinations have not been shown for improved readability.

A similar approach can be applied to the definition of the individual score for generalized interolog (cross-species) predictions. In this case, the dependence of functional distance upon sequence dissimilarity of two proteins from different organisms can be described in a similar way with the assumption that all pre-defined orthologs have identical functions and the limitation that only proteins which have orthologs in both organisms are used for the calculation of functional distance. These conditions allow us to calculate the functional distance between protein Ax from organism X and Ay′ from organism Y, where Ay is the only true ortholog of Ax and Ay is a paralog of Ay′. Their functional distance should be equal to the probability to not observe an interaction between proteins Ay′ and By in organism Y, given that the protein Bx, which is the only true ortholog of By from organism X interacts with a protein Ax′ paralogous to the protein Ax. The value for functional distance averaged over all possible B’s can then be plotted as a function of the sequence dissimilarity between Ax and Ay′, which is equal to the 1– sim(Ax, Ay′), where sim(Ax, Ay′) is amino acid sequence similarity. After the same normalization procedure used for interparalogs, the resulting dependency for pairwise comparisons has the form shown in Figure 4.

Figure 4 illustrates a slightly modified formula (3) that is also a reasonable approximation for the prediction of interolog interactions. The fit appears to be especially good when we take into account that there is a rather small data portion containing protein pairs with the similarity >0.7–0.8 in different organisms. One can estimate the probability for A′ to keep the same interactions as its ortholog A from another organism, using the modification of the empirical formula (3):

(6) $$\begin{array}{ll} F_{\text{A}{\text{A}}^{\prime }}\;= & 1-p_{\text{A}{\text{A}}^{\prime }}\;\approx \;-\;(\text{Q}/\text{c})*\tau \;*arctg\;(-\text{T}/\tau )\\ & +\;\text{Q}*\;\tau *\;arctg\;((\text{x}(\text{A},\;{\text{A}}^{\prime })-\text{T})/\tau ) \end{array}$$

where c is an additional adjustment reflecting the fact that even very similar proteins from different organisms still can have different functions.

The exact estimation of all pairwise fitting parameters is impossible for some organism pairs due to the small amount of data. It can be shown, however, that the main coefficient T for all well-studied organism pairs varies from 0.4 to 0.6, as compared to the range [0.2, 0.33] found for interparalogs. To better estimate the parameters, we created a dataset of averaged over all organism pairs and determined all coefficients for this dataset, which are:

(7) $$\begin{array}{l} \text{Q}\;=\;0.4031\\ \text{T}\;=\;0.5259\\ \tau \;=\;0.1237\\ \text{c}\;=\;0.07151 \end{array}$$

These parameters were used for calculation of the individual score using the same formula (5) that we used for interparalogs.

### Definition of the combined score

Individual scores must be added into one “combined” score for every predicted interaction. The “combined” score must take into account the fact that there could be several interactions in the training sets, which lead to the same prediction in the target organism. In previous work, the combined scores were typically defined as trivial functions such as minimum, maximum, sum, or average, depending on the nature of the individual score (Yu et al. 2004). However, these approaches do not improve the score with the increase in the number of individual interactions in the training set that predict the scored interaction. Here, we present a probabilistic way to calculate the combined prediction score as a function of individual scores.

Because the individual score can be interpreted as an independent probability, the final interaction probability can be expressed as:

(8) $${\text{M}}_{\text{fin}}\;=\;1-\prod \left(1-p_{i}\right);$$

where (1–*p_i_*) is the probability that the target proteins do not interact in every prediction method. We will refer to the score calculated using this formula as the “M-score.”

### Definition of the protein family size

**Figure 5.**
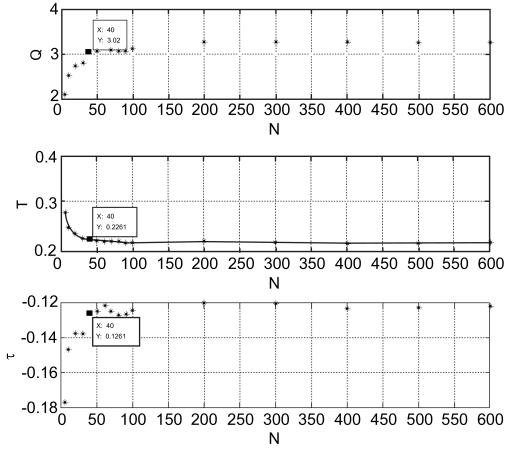
Parameters of the Kapitza fit (3) for the dependency between the average “functional distance” *F* AA′ and “sequence dissimilarity” between paralogous proteins for *Homo sapiens* network, where for each possible N, only protein families with the number of proteins ≤N have been taken. Here, for each N, three types of coefficients (Q, T,τ) have been defined with 95% confidence bounds.

The M-score depends on the total number of individual predictions that are taken into account for the formula (8). We found that taking all possible prediction events for the M-score was disadvantageous for several large protein families. Proteins in these families have small values for sequence similarity between them. However, adding numerous predictions with weak individual scores distorted the statistics. We have resolved this problem by restricting the maximum allowed protein family size and making all protein families no larger than N, which is the characteristic protein family size. To define N, we calculated the minimum family size that would not significantly change the statistical properties of the predicted network. We have monitored how the fitting parameters change with the maximum allowed family size, thus using them as network properties. The results of this investigation are shown in Figure 5. One can see that for Ns larger than 40, all parameters stop changing significantly. The maximum allowed family size of 40 is easy to understand intuitively, since there is no known protein family with such a large number of members. Therefore, we selected this value to limit the number of homologs taken into account for M-score calculation.

## Evaluation of Prediction Quality

To evaluate the quality of our prediction method, we have chosen one article, which describes tasks and methods similar to ours, and has an online supplementary data with the predictions (Yu et al. 2004). We have not compared our results with other published prediction methods either because their prediction results were not available publicly or because they rely on more than just experimental interactions and sequence similarities. Our goal, however, was to develop a prediction method that used exclusively the sequence similarity information as an input because it is the only type of information available for newly sequenced genomes.

We have looked at the percentage of true positives for the top 1,000 predictions. Our values were compared with those obtained by the method, described in (Yu et al. 2004). The score used in this paper was the “maximum of joint similarity” (J-score) value:

(9) $${\text{J}}_{\text{fin}}\;({\text{A}}^{\prime },\;{\text{B}}^{\prime })\;=\;{\text{max}}_{\text{i}}\;[\surd (\text{sim}\;({\text{A}}_{\text{i}},\;{\text{A}}^{\prime })*\;(\text{sim}({\text{B}}_{\text{i}},\;{\text{B}}^{\prime })]$$

We have defined a percentage of true positives as the number of prediction pairs, which were present in the “golden positive set” (Table 1).

For the best-studied networks (human and yeast), the negative sets are readily available. We used the Receiver Operating Characteristics (ROC) curve that plots a true positive rate as a function of false positive rate and is normally used to evaluate the accuracy of a classification score. The accuracy of methods is measured by the area under the ROC curve (Table 1, auROC values).

One can see that the M-score (8) provides a significantly better true positive rate than the joint similarity J-score (9) for the prediction of interparalogs. For example, 35% of the top 1,000 yeast interparalog predictions scored with the M-score can be confirmed by the “golden positive set”, while the same number of top predictions scored with the J-score can be confirmed in only 20% of all cases. The same values for the human network are 20% and 10% for M-score and J-score, respectively. Table 1 also shows that the M-score has better classification strength than the J-score as judged by the area under the ROC curve (auROC).

To define the set of the most reliable predictions, we have calculated the optimal M-score cutoff values using the percentage of verified interactions as a function of the score cutoff (*data not shown*). Using these estimated M-score cutoffs (Table 2), we have predicted the most reliable for 20,000 human, 12,000 yeast, 3,500 worm, 2,500 arabidopsis and 2,200 fly interactions.

A similar procedure can be used to evaluate interolog predictions. In this case, it is possible to combine M-scores from different organism pairs in the same manner as it is done for interparalogs from one organism using the formula (8). We found that the quality of interologs prediction using multiple organism pairs was about the same as the joint similarity method (*data not shown*). Thus, it appears that the prediction based on the interactions from multiple organisms has no advantages over the prediction based on an interacting pair from only one organism. We can conclude this because the protein function is conserved less between different genomes than it is for paralogs. Alternatively, it may mean that the knowledge about interolog interactions from several organisms is highly redundant and does not increase the statistical power of the prediction. We discuss this observation in more detail in the next section.

The proposed scoring system includes three different techniques: (a) calculation of individual score, (b) combining the individual scores and (c) finding of protein family size. In order to evaluate an extent of contribution for each technique we have studied different score schemas for yeast and human interparalogs. The following scores were compared

- “J-score”—formula (9);
- “modified J-score”, where (sim(A_i_, A′)*(sim (B_i_, B′)) was replaced by the *p_i_* (*A*′*, B*′) value from formula (5);
- “unrestricted M-score”—formula (8), without protein family size restrictions;
- “M-score”—formula (8), where the maximum allowed protein family size is set to 40;

The performance of J-score and “modified J-score” is about the same in every organism, since the dependence of *p_AiA′_* value (formula (3)) on similarity value sim(A_i_,A′) is monotonous. Since it is necessary to have the probabilistic individual score in order to combine them into M-score, the performance of techniques a) and b) should be considered together. In case of yeast interparalogs the performance of “M-score” and “unrestricted M-score” are the same, since there are no large protein families (>40) in yeast and protein family size restriction does not change anything. Hence, for the yeast interparalogs the only difference is between “J-score” and “M-score” (Table 1), and the main technique, which improves the quality of predictions is b) - the combination of individual scores.

The relative contribution of different techniques changes dramatically in case of predictions for human proteins. The restriction on protein family size improves the quality of predictions significantly. A percentage of true positives for top 1000 “J-score”, “unrestricted M-score” and “M-score” predictions are 10%; 12% and 20% respectively. The same values for top 2000 are 9%; 10% and 18%. For top 5000 the numbers are 8%; 9% and 13% (Table 1). This suggests that 80% of M-score success in human interparalog predictions depends on restriction of protein family size (technique c), whereas 20% on combining the individual scores (technique b) and the formula for calculation of individual score (technique a).

## Discussion

We have developed a novel scoring system that improves the reliability of predictions about protein physical interactions using the information about known interactions of their homologs. The method takes into account not only the sequence similarity between homologs but also the number of known interactions for different homolog pairs. On average, it assigns better scores to interactions predicted on the basis of several “hits,” as compared to single-hit predictions. We show that this approach allows more reliable prediction of interactions using paralogous proteins.

Individual scores used in our algorithm are based on empirical estimates of the likelihood that a pair of homologous proteins with a specific sequence similarity shares a common interaction partner. We show that the correlation between this probability and a sequence similarity is approximated best by the formula developed to describe the self-similar growth of a population with a finite reproduction lifespan. The formula can be used, albeit with different parameters for prediction of both interologs and interparalogs. We found, however, that our method performs better for interparalog predictions while, for interolog predictions, its performance is comparable with the joint similarity score. The formula was developed as a best fit for human population growth by S.P. Kapitza. The curve is supposed to fit the explosive growth in the beginning that changes to significant slowdown after critical time T. In the case of protein functional divergence, this phenomenon means that proteins start losing their functional similarity more slowly after their sequences diverge beyond critical similarity. One can speculate that the far-diverged proteins still must share common interactions in order to continue being functional. However, a closer look at Figure 1 reveals that the number of common partners drops below 20% beyond the critical sequence similarity (functional distance >0.8), which makes the importance of this observation negligible.

The analysis of parameter T, corresponding to the critical point in the functional distance, suggests the difference between functional divergence of interologs and interparalogs. This critical point corresponds to the point in time in which population growth begins to slow down following the explosive phase. While the characteristic value of T for interparalogs lies between 0.2 and 0.3 and corresponds to the sequence similarity 0.7–0.8, these numbers for interologs are 0.4–0.6. This observation suggests that two paralogs begin to lose functional similarity as measured by the number of their common interaction partners when their sequence similarity drops below 70–80%. The interologs, on the other hand, remain functionally similar until their sequence similarity remains above 40–60%. This conclusion is intuitively clear: unless a paralog acquires the new function relatively quickly during the sequence divergence, it will probably be lost from the genome due to the loss-of-function mutation. Interologs, however, are constantly under evolutionary pressure to maintain the function during a rather long time of divergence.

We have found that the quality of the interolog prediction does not benefit from combining knowledge about interactions from multiple organisms. Because our own data suggest that interologs remain functionally similar during a longer divergence period, the most likely explanation is that information gathered from multiple organisms is redundant; i.e. it is enough to know an orthologous interaction pair in one organism in order to predict an interolog with high confidence. Therefore, the prediction of physical interaction could be done by selecting only one best predictive organism. Our data also confirm the observation made in (Mika and Rost, 2006) that the interolog predictions are less reliable than interparalog predictions.

## Figures and Tables

**Table 1.** Evaluation of Interparalog Predictions.

f(x) = –Q*t*atan(–T/t) + Q*t*atan((x–T)/t)	**Human**	**Yeast**	**Worm**	**Arabidopsis**	**Fly**
Q	3.068	3.096	3.672	2.134	4.515
T	0.2234	0.2443	0.2794	0.1747	0.3305
t	−0.1248	0.1284	0.1018	0.2293	0.08121
**% verified (top 1,000, M-score)**	**20%**	**35%**	**11%**	**45%**	**35%**
% verified (top 1,000, J-score)	10%	20%	7%	25%	30%
**% verified (top 2,000, M-score)**	**18%**	**30%**	**12%**	**34%**	**24%**
% verified (top 2000, J-score)	9%	17%	8%	25%	22%
**% verified (top 5000, M-score)**	**13%**	**28%**	**11%**	**28%**	**16%**
% verified (top 5000, J-score)	8%	15%	6%	24%	14%
M-score auROC*	**0.864**	**0.815**	**-**	-	-
J-score auROC*	0.855	0.808	-	-	-

* Accuracy of methods is measured by the area under the ROC curve—auROC value.

**Table 2.** Predictions.

	**Human**	**Yeast**	**Worm**	**Arabidopsis**	**Fly**
Interparalog cutoff	0.8	0.26	0.26	0.8	0.8
# of interparalog predictions with this cutoff	~20000	~12000	~3500	~2500	~2200
% verified interparalogs (cutoff)	10%	30%	12%	32%	23%
generalized Interolog cut-off	0.01	0.01	0.01	0.01	0.01
# of generalized interolog predictions with this cutoff	~75000	~9000	~55000	~100000	~51000
% verified generalized interologs (cutoff)	4%	57%	5%	9%	3%
